# Gun violence against unhoused and unstably housed women: A cross-sectional study that highlights links to childhood violence

**DOI:** 10.1186/s40621-021-00348-4

**Published:** 2021-08-19

**Authors:** Rose M. C. Kagawa, Elise D. Riley

**Affiliations:** 1 Violence Prevention Research Program, Department of Emergency Medicine, School of Medicine, University of California at Davis, 2315 Stockton Blvd., Sacramento, CA 95817 USA; 2 Division of HIV, Infectious Disease and Global Medicine, Department of Medicine, University of California, San Francisco, 1001 Potrero Ave, San Francisco, CA 94110 USA

**Keywords:** Violence, Gun violence, Homelessness, Women

## Abstract

**Background:**

Unstably housed women experience high levels of violence. While previous studies have investigated psychological, physical, and sexual violence, weapon and gun violence are rarely delineated. We examined factors associated with experiencing violence as an adult among unhoused and unstably housed women, with a focus on gun violence.

**Methods:**

We recruited women with a history of housing instability from San Francisco homeless shelters, street encampments, free meal programs, low-income hotels, and health clinics. Participants completed interviews including questions regarding both childhood and adult violence. We used multivariable logistic regression to examine associations of specific types of childhood violence (i.e., physical, sexual, gun, other weapon-involved violence), age, race, ethnicity, educational attainment, and sexual orientation, with risk of experiencing specific types of violence as an adult (i.e., gun, other weapon-involved, physical violence). Because the violence outcomes were not rare, odds ratios were transformed to approximate risk ratios.

**Results:**

Nearly half of women (n = 110, 45%) had been attacked with a gun as an adult. Violent victimization in adulthood was common, with 33% having experienced all three forms of violence. The probability of being attacked with a gun as an adult was almost 70% higher among women who had been hit or kicked hard enough to cause injury as a child (RR = 1.68; 95% CI = 1.24, 2.11), and this association was present for all three violence outcomes. No other risk factors explored, including other types of violence experienced as a child, met the statistical threshold to be considered significant.

**Conclusions:**

The high prevalence of gun violence reported here is consistent with prior studies of violence conducted in similar populations. Considered in combination with prior research, findings suggest multi-generational violence prevention interventions for low-income women and girls are needed. They also emphasize the need to more actively include people who experience unstable housing in statewide and national violence surveys to better understand the extent of the problem and address on a broader scale the high rates of violence experienced by unstably housed populations, which have been consistently documented in local research.

## Background

Women living in unstable housing conditions experience violence at rates far exceeding the national average, and unhoused women in particular are at great risk of physical and sexual assault (Wenzel et al. 2006; Nyamathi et al. 2000). Studies estimate approximately a third of unstably or unhoused women have experienced violence that could lead to injury (Nyamathi et al. 2000; Wenzel et al. 2001) and serious mental health consequences (Tsai et al. 2015), with a far higher prevalence among unhoused women living outside of a shelter or treatment facility (Nyamathi et al. 2000; Wenzel et al. 2000). Among studies that report violence against unhoused and unstably housed women, few report specifically on weapon-involved violence. Among those that have, a study of San Francisco women living in unstable housing conditions found 48% had experienced physical violence without a weapon and 18% had experienced physical violence with a weapon in the six months prior to interview (Riley et al. 2020). Factors specifically associated with gun and other weapon-involved violence against unstably housed women are unclear.

Risk of violent victimization is intimately woven into the causes, conditions, environments, and consequences of homelessness (Knight et al. 2014). Child abuse and neglect and the early life environments that are associated with child abuse (Merritt 2009) may increase risk for homelessness in adolescence (Hyde 2005) and later in life (Wenzel et al. 2001; Sullivan et al. 2000). Domestic violence is an immediate cause of homelessness, particularly for women who escape violent households into temporary living situations (Zorza 1991). However, research also suggests that, among women experiencing homelessness or housing instability, violence is most often perpetrated by people other than primary partners (e.g. strangers, ex-boyfriends, neighbors) (Riley et al. 2014). While the contexts in which this violence takes place are extremely varied (Jasinksi et al. 2005), violence and trauma are especially common where women live and spend time in their day-to-day routines (Knight et al. 2014; Jasinksi et al. 2005). Additionally, the conditions of being unhoused may lead people to engage in higher risk behaviors (e.g. trading sex for money, panhandling) in order to meet their basic needs, which puts them at further risk for violent victimization (Wenzel et al. 2001). The experience of violence in this population is both common and complex in terms of its causes, correlates and consequences.

Violence involving weapons, and particularly firearms, is considered a severe form of violence (Kagawa et al. 2020). Previous research has documented higher rates of violence resulting in injury and other “major physical violence” among unstably housed women as compared to housed women (Wenzel et al. 2006), and another study estimated 45% of young adults experiencing homelessness had been victimized with a firearm (Hsu et al. 2020), but little research has explored exposure to weapon-involved violence specifically in this population. In addition, some influential nation-wide assessments of gun violence involve household surveys, which systematically exclude persons experiencing homelessness (Planty and Truman, 2013). Understanding the prevalence of exposure to gun and other weapon-involved violence as well as risk factors for this exposure is important for gaining a more comprehensive understanding of the experience of violence in this population. The objective of this study was to identify characteristics and life events that are associated with increased risk for experiencing gun violence, violence involving other weapons, and physical violence within this already high-risk population. Extending prior violence-related research in this population (Wong et al. 2016), we hypothesized that gun violence experienced as a child is associated with a greater likelihood of having experienced gun violence as an adult.

## Methods

### Study design

The study described here is a sub-study within “Polysubstance Use and Health Outcomes Evaluation” (PULSE), a prospective study of unstably and unhoused women living in San Francisco. Data were collected between June 2016 and January 2019 on a range of demographic, socioeconomic, personal and family history, and health-related topics. The current study relies solely on data collected at baseline as questions about experiencing violence were not repeated.

### Study participant recruitment

Recruitment methods were based on those developed in prior studies to obtain a sample that reflected the larger population of women experiencing homelessness in San Francisco (Riley et al. 2014; Burnam and Koegel 1988). Individuals were recruited from shelters, free meal programs, low-income single room occupancy (SRO) residential hotels, and street encampments.

### Study sample

Inclusion criteria included female sex at birth, age ≥ 18 years, and a history of housing instability (i.e., slept in public or a homeless shelter, or stayed with a series of associates because there was no other place to sleep [“couch-surfed”]). HIV-positive persons were oversampled to accomplish HIV-related aims of the original study. Previous research suggests these participants were similar to HIV-negative participants across a range of measured variables (Riley et al. 2020). Participants were remunerated $40 for each study interview. All study procedures reported here were approved by the Institutional Review Board at the University of California, San Francisco.

### Study measures

We examined three study outcomes, including whether the individual had experienced an attack with a gun, with another weapon, or with physical violence, as an adult. These experiences were determined using the following three survey questions: “As an adult, were you ever attacked with a gun?”, “As an adult, were you ever attacked with a knife, or other weapon?”, and “As an adult, were you ever hit or kicked hard enough to be injured?”

Primary exposures examined included four separate childhood trauma events: “As a child, were you ever attacked with a knife, or other weapon?”, “As a child, were you ever attacked with a gun?”, “As a child, were you forced or made to have sexual contact?”, and “As a child, were you hit or kicked hard enough to be injured?” Additional exposures considered as potentially associated with having experienced each form of adult violence included demographics (age (continuous), race (Alaskan/Native American, Asian/Pacific Islander, Black, white, and multiracial or other), and ethnicity (Latina)), highest education level completed (less than high school, high school or GED completed, college graduate), sexual orientation (homosexual, heterosexual, bisexual, other), and time spent homeless (years). Race and ethnicity were measured using self-report and reflect the experience of being a particular race/ethnicity (e.g. experiencing racism) rather than a biological or genetic construct (Schwartz 2001). Results shown use white, heterosexual, and completed high school as the reference groups for their respective variables. However, regressions were repeated to allow for each racial group, each sexual orientation, and each education level to serve as the reference group. Finally, while additional variables were of interest (e.g. social support), these reflected each woman’s status at the time of the interview and thus were subsequent to any experienced violence.

### Analytic approach

We used multivariable logistic regression models in which experiencing an attack as an adult was the dependent variable. While the study used only data collected at one time point, most risk factors considered would be present before adult trauma, and we therefore refer to risk of weapon-involved violence as an adult. Because the outcome was not rare, we transformed the statistically significant odds ratios to risk ratios following Zhang and Kai (1998) to aid in interpretation, and refer to the risk ratios in discussion. The formula is as follows:

$$RR\approx \frac{OR}{\left(1-{p}_{u}\right)*({p}_{e}*OR)}$$

where RR is the approximate risk ratio, OR is the odds ratio, and ${p}_{u}$ and ${p}_{e}$ are the prevalence of the outcome among the unexposed and exposed, respectively.

For each risk factor, we attempted to control only for variables we could reasonably assume preceded the exposure of interest. For this reason, associations with age, race/ethnicity, sexual orientation, and traumatic childhood events were read from the model including only these variables. Educational attainment was added to and read from a separate model as it could be considered a mediator in the aforementioned relationships. Time spent homeless was added to and read from yet another model as we could not determine from the data whether the bulk of time spent homeless preceded or followed the violent events of interest; this analysis was considered as exploratory. Separate models were run for each outcome: being attacked with a gun, being attacked with a knife or another weapon, and being hit or kicked hard enough to cause injury. Missing data were treated as missing completely at random and not included in corresponding analyses.

## Results

The response rate during recruitment activities was 89%, which resulted in our recruitment of 245 women (77 from low-income residential hotels, 70 from free meal programs, 50 from the Ward 86 HIV clinic, 44 from homeless shelters and 4 from street encampments). Study participants were between the ages of 24 and 75 (mean = 51.6 years) (Table 1); 39.6% were black, 27.8% were white, and 22.9% were multiracial or “other”. For most (56.8%), the highest level of education completed was high school or GED, 66.9% were single, and 73.8% were heterosexual. On average, study participants had experienced 8.1 years unhoused or unstably housed (range: < 1 to 37). Of the 245 women surveyed, three (1.2% of the sample) were missing data on childhood gun violence, three (1.2%) were missing data on childhood sexual violence; and two (0.8%) were missing data on childhood physical violence. Because some women were missing responses to more than one variable, adjusted analyses excluded six total participants (2.4%).

**Table 1:** Sample characteristics (N = 245)

Variable	N (%)
Age	51.6 years (SD = 10.8)
*Race*	
Black/African American	97 (39.6)
Alaskan/Native American	16 (6.5)
Asian/Pacific Islander	8 (3.3)
White	68 (27.8)
Multiracial/Other	56 (22.9)
Hispanic/Latina	37 (15.1)
*Sexual orientation*	
Heterosexual	181 (73.9)
Homosexual/Lesbian	16 (6.5)
Bisexual	39 (15.9)
Other	9 (3.7)
Hit or kicked as child	119 (48.6)
Forced sexual contact as child	108 (44.1)
Attacked with gun as child	19 (7.8)
Attacked with knife or another weapon as child	51 (20.8)
*Education level*	
Less than high school	72 (29.6)
Completed high school	138 (56.8)
College graduate	33 (13.6)
Time homeless	8.1 years (SD = 7.8)

**Fig. 1:**
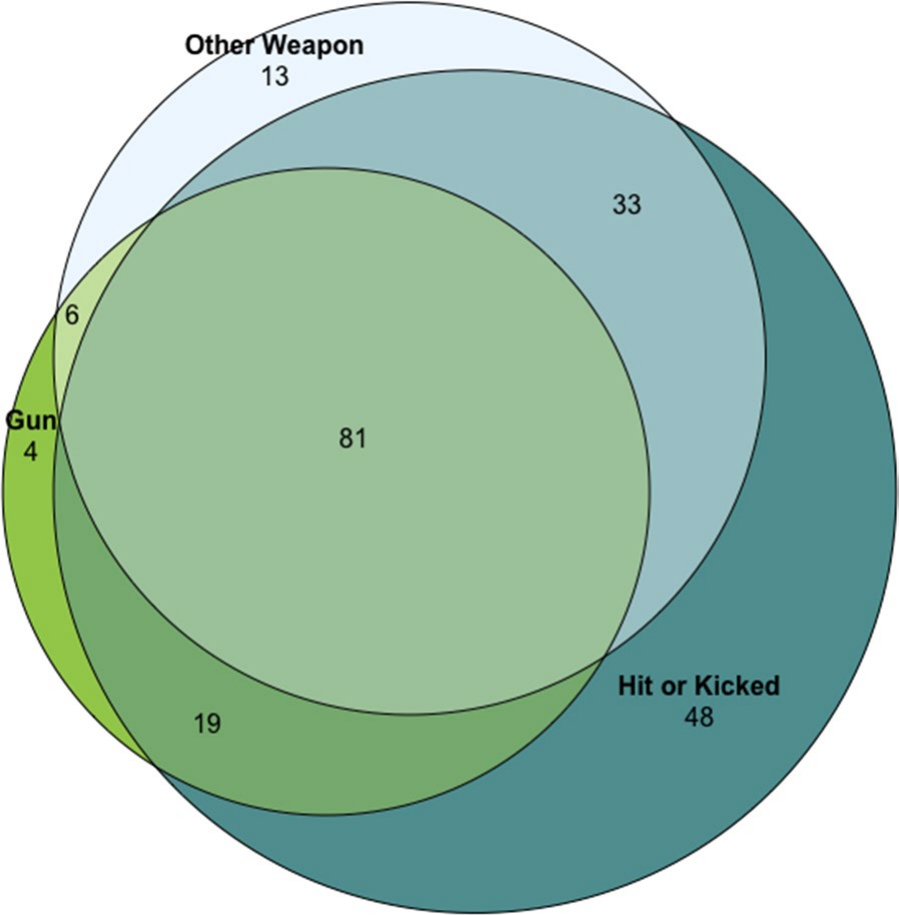
Overlapping exposure to three forms of violence: gun, other weapon, hit or kicked hard enough to cause injury (counts shown, N = 245). 41 participants did not experience any of the three forms of violence presented here

Forty-five percent of respondents (n = 110; 95% CI = 38.6–51.2%) had been attacked with a gun as an adult, 54.3% (n = 133, 95% CI = 48.0–60.6%) had been attacked with a knife or other non-gun weapon, and 73.8% (n = 181, 95% CI = 68.3–79.4%) had been physically attacked (i.e., hit or kicked hard enough to cause injury). Most women who experienced one form of violence had also experienced other forms of violence in adulthood (Fig. 1); among those attacked with a gun, 90.9% had been hit or kicked hard enough to cause injury and 79.1% had been attacked with a knife or another weapon as an adult. In fact, 33.1% (n = 81) of women had experienced all three forms of violence, as compared with 16.7% (n = 41) of women who had not experienced any of the three forms as adults.

The prevalence of experiencing any of the three forms of adult violence did not differ, in a statistically significant manner, across the social or demographic variables under study with one exception. Women of Hispanic/Latina ethnicity were more likely than those not of Hispanic/Latina ethnicity to report experiencing gun violence as an adult (59.5% vs. 42.3%; *p* = 0.053).

A greater percentage of the women who had been hit or kicked hard enough to cause injury as children, compared with those who had not, reported experiencing violence as an adult (56.3% vs. 33.9% reported adult gun violence, 67.2% vs. 41.9% reported adult other weapon-involved violence, 86.6% vs. 61.3% reported adult physical violence; all *p* < 0.05 using Pearson’s chi-squared tests) (Table 2). Women who experienced other forms of childhood violence—being attacked with a gun, being attacked with a knife or another weapon, and forced sexual contact—also tended to have a higher likelihood of experiencing some form of adult violence.

**Table 2:** Prevalence of experiencing violence with a gun or another weapon, or being hit or kicked hard enough to cause injury as an adult among unhoused and unstably housed women who experienced specific forms of childhood violence

	% Attacked with Gun as Adult (n of N); p-value	% Attacked with Knife or Another Weapon as Adult (n of N); p-value	% Hit or Kicked Hard Enough to Cause Injury as Adult (n of N); p-value
Hit or kicked as child versus not	56.3% (67 of 119) versus 33.9% (42 of 124); < 0.001	67.2% (80 of 119) versus 41.9% (52 of 124); < 0.001	86.6% (103 of 119) versus 61.3% (76 of 124); < 0.001
Forced sexual contact as child versus not	48.2% (52 of 108) versus 41.04% (55 of 134); 0.269	58.3% (63 of 108) versus 50.0% (67 of 134); 0.196	80.6% (87 of 108) versus 68.7% (92 of 134); 0.036
Attacked with gun as child versus not	68.4% (13 of 19) versus 42.2% (94 of 223); 0.027	79.0% (15 of 19) vs 51.6% (115 of 223); 0.022	89.5% (17 of 19) versus 72.2% (161 of 223); 0.101
Attacked with knife or another weapon as child versus not	56.9% (29 of 51) versus 41.8% (81 of 194); 0.054	68.6% (35 of 51) versus 50.5% (98 of 194); 0.021	84.3% (43 of 51) versus 71.1% (138 of 194); 0.057

*P* values presented are from Pearson’s Chi-squared tests and comparisons are drawn between those who reported experiencing the type of violence listed as a child and those who did not report that specific form of violence as a child

Missing data: 3 people are missing data on childhood gun violence and 3 are missing data on childhood sexual violence; 2 people are missing data on childhood physical violence

In adjusted analyses, the probability of being attacked with a gun as an adult was 68% higher among women who had been hit or kicked hard enough to cause injury as a child compared to women who did not report being hit or kicked as a child (OR = 2.53 [95% CI = 1.399, 4.571] ≈ RR = 1.68 [95% CI = 1.237, 2.106]). A similar association was observed for the other two outcomes: attacked with a knife or other weapon in adulthood (OR = 2.55 [95% CI = 1.423, 4.568] ≈ RR = 1.56 [95% CI = 1.213, 1.855]) and hit or kicked hard enough to cause injury in adulthood (OR = 3.49 [95% CI = 1.736, 7.022] ≈ RR = 1.38 [95% CI = 1.195, 1.493]) (Table 3). The only other variable tested to reach statistical significance was education level. Women with a college or graduate degree were more likely than women who completed their education with a high school degree to report being attacked with a knife or another weapon as an adult (OR = 2.78 [95% CI = 1.100, 7.052] ≈ RR = 1.46 [95% CI = 1.047, 1.733]) (Table 3).

**Table 3:** Risk factors for experiencing gun violence, other weapon-involved violence, or physical violence as an adult among unhoused and unstably housed women (N = 239)

Variable	Attacked with Gun Adjusted Odds Ratio (95% CI)	Attacked with Knife or Another Weapon Adjusted Odds Ratio (95% CI)	Hit or Kicked Hard Enough to Cause Injury Adjusted Odds Ratio (95% CI)
*Model 1*			
Age	1.01 (0.987, 1.040)	1.00 (0.979, 1.030)	0.99 (0.964, 1.021)
*Race*			
Alaskan/Native American	1.19 (0.366, 3.896)	0.89 (0.274, 2.858)	2.31 (0.534, 9.972)
Asian/Pacific Islander	0.77 (0.159, 3.752)	0.59 (0.120, 2.873)	1.56 (0.257, 9.446)
Black/African American	0.98 (0.493, 1.942)	1.25 (0.642, 2.451)	1.48 (0.708, 3.081)
Hispanic/Latina	1.47 (0.637, 3.391)	1.12 (0.474, 2.665)	0.74 (0.281, 1.927)
Multiracial/Other	1.67 (0.728, 3.830)	2.08 (0.888, 4.858)	1.81 (0.688, 4.737)
White	Ref	Ref	Ref
*Sexual orientation*			
Homosexual/Lesbian	0.36 (0.107, 1.230)	1.03 (0.333, 3.171)	1.44 (0.367, 5.639)
Bisexual	0.61 (0.274, 1.338)	1.13 (0.513, 2.491)	1.06 (0.418, 2.667)
Other	0.64 (0.135, 3.018)	1.44 (0.293, 7.093)	0.52 (0.100, 2.750)
Heterosexual	Ref	Ref	Ref
Hit or kicked as child	2.53 (1.399, 4.571)	2.55 (1.423, 4.568)	3.49 (1.736, 7.022)
Forced sexual contact as child	0.86 (0.464, 1.600)	0.84 (0.452, 1.551)	1.06 (0.523, 2.140)
Attacked with gun as child	2.39 (0.768, 7.440)	2.72 (0.750, 9.891)	1.87 (0.352, 9.911)
Attacked with knife or another weapon as child	1.29 (0.600, 2.754)	1.30 (0.596, 2.834)	1.44 (0.548, 3.804)
*Model 2*^^^			
*Education level*			
Less than high school	1.40 (0.740, 2.653)	1.00 (0.527, 1.898)	0.64 (0.312, 1.307)
Completed high school	Ref	Ref	Ref
College graduate	0.73 (0.300, 1.753)	2.78 (1.100, 7.052)	1.90 (0.653, 5.538)
Model 3*			
Time homeless	1.03 (0.995, 1.076)	1.04 (0.998, 1.084)	1.05 (1.000, 1.112)

All models include age, race, ethnicity, sexual orientation, and each of the four childhood trauma variables shown

Missing data: 3 women are excluded from the analysis due to missing data for childhood gun violence, an additional 2 women are excluded from the analysis due to missing data for childhood sexual violence, and an additional 1 woman is excluded from the analysis due to missing data on childhood physical violence

^^^Model 2 additionally includes educational attainment. (2 additional women are excluded from the analysis due to missing data on education level; N = 237)

*Model 3 additionally includes time spent homeless. (2 additional women are excluded from the analysis due to missing data on time unhoused; N = 237)

In exploratory analyses, greater time spent homeless was positively associated with having experienced all three forms of violence. Each additional year spent homeless was associated with an average increase in risk of having been attacked with a gun as an adult of 3% (OR = 1.03 [95% CI = 0.995, 1.076] ≈ RR = 1.03 [95% CI = 0.995, 1.076]), for having been attacked with a knife or another weapon as an adult of 4% (OR = 1.04 [95% CI = 0.998, 1.084] ≈ RR = 1.04 [95% CI = 0.998, 1.084), and having been hit or kicked hard enough to cause injury as an adult of 5% (OR = 1.05 [95% CI = 1.000, 1.112] ≈ RR = 1.05 [95% CI = 1.000, 1.112]).

## Discussion

This study documents a high prevalence of severe violence experienced as an adult in a population of unstably housed and unhoused women living in an urban environment. Nearly half (44.9%) of study participants had been attacked with a gun and over 80% had experienced at least one episode of being attacked with any weapon (i.e., a gun or other weapon), or being hit or kicked hard enough to cause injury. Many women (33.1%) had experienced all three forms of violence in adulthood. Latina women were more likely than non-Latina women to experience gun violence as an adult, and more time spent homeless was associated with experiencing all three forms of violence as an adult. In adjusted analyses, only childhood physical violence was associated, strongly and consistently, with an increased risk of experiencing violence as an adult. Having a college degree or higher, compared with having a high school degree, was also associated with an increased risk of violence, but only for violence involving a knife or another weapon. Results suggest that unhoused and unstably housed women experiencing a single type of violence as an adult have likely experienced other forms of violence, and this risk of violence is associated with experiencing interpersonal physical violence in childhood.

The current study builds on our previous research, which indicates that violence against homeless and unstably housed women is common (Riley et al. 2014), leads to severe mental health consequences (Tsai et al. 2015), and is perpetuated in part by entrenched social norms and reinforcing environments that normalize violence (Knight et al. 2014). It also builds on the larger body of existing evidence, which consistently links violence against unhoused and unstably housed adult women to multiple violence types (Wenzel et al. 2006; Riley et al. 2014), prior childhood violence (Wong et al. 2016), mental health conditions (e.g., depression, anxiety, PTSD) (Duke and Searby 2019; Salem et al. 2020), substance use (Kitson 2020), unmet subsistence needs (e.g., housing and food insecurity) (Riley et al. 2014), poor social support (Wenzel et al. 2004), barriers to care (Bassuk et al. 1972; Hatton 2001), and an insufficient societal response (Beijer et al. 2015). Here we show that 45% of unstably housed women not only experience physical violence, but a form of violence with great lethal potential, gun violence.

Our data did not distinguish between very recent violence and all violence experienced as an adult, leaving us unable to identify proximal risk factors for adult-experienced gun violence. However, our prior work, which did distinguish very recent violence, but did not differentiate between gun violence and all physical violence, showed that recent physical violence against unhoused and unstably housed women was significantly associated with unmet subsistence needs (i.e., insufficient access to housing, food, clothing or hygiene needs).

Studies characterizing violence against women who experience housing instability have shown consistent results over the past 20 years, yet it remains a problem. Moreover, surveillance that quantifies the problem outside of research, and interventions that target this very high-risk group are still lacking. Larger-scale efforts may be more effective if they target multiple generations for both adult-experienced violence and childhood violence. Implementation of both will require attention to unmet subsistence needs and deprivation. For the purposes of understanding the full extent of the problem, our research suggests that state-wide and national surveys regarding exposure to and consequences of violence experienced in the U.S. should actively include unstably housed and unhoused individuals (Lemieux and Felson 2012). Given the disproportionately high rate of gun violence among Latina women found here, such larger-scale efforts should include outreach to Hispanic/Latinx communities.

The association between experiencing physical violence as a child and all three forms of violence as an adult mirrors research on the long-lasting effects of childhood victimization and its association with future violence experienced as an adult (Parks et al. 2011; Banyard et al. 2000; Coid et al. 2001; Roodman and Clum 2001). This study, like much of this literature, lacks early-life measures of risk factors for violence experienced as a child or as an adult, such as family poverty and parental substance use, which may confound the observed association. As such, we cannot disentangle the specific role of being hit or kicked hard enough to cause injury from the environments that often surround childhood violence. Experiencing maltreatment as a child is associated with a range of factors that put children at risk for continuing to experience or be involved in violence (Ferrara et al. 2016). Child maltreatment is also a risk factor for housing insecurity in adulthood (Schuck and Widom 2021). Regardless of the specific causal mechanisms, these findings reinforce the importance of early life intervention among children experiencing violence. Related research suggests comprehensive approaches such as home-visiting programs for parents of very young children (Moffitt and Klaus-Grawe 2013; Coore Desai et al. 2017), financial assistance (Palmer et al. 2019), affordable housing (Freedman and Owens 2011), and other family supports (Moffitt and Klaus-Grawe 2013), could relieve immediate hardships in early life and may contribute to the prevention of harmful sequelae into adulthood.

The experience of violence as an adult was also associated with the amount of time spent unhoused. However, we were unable to determine the direction of the relationship (i.e. whether the experience of violence contributed to homelessness or whether homelessness made experiencing violence more likely), and context specific bi-directionality is possible. Unstable housing is both a cause and consequence of violence perpetration and victimization (Wenzel et al. 2001; Hyde 2005; Zorza 1991). The literature suggests many of the factors that lead to homelessness also make finding and maintaining a safe and stable home difficult (Baker et al. 2010); for example, women escaping an abusive relationship may, partly as a result of the abusive relationship, have a history of evictions, poor credit, a criminal record, limited income potential, psychological trauma and other barriers that prevent her from securing or maintaining housing (Baker et al. 2010). Coupled with a short supply of affordable housing stock, rental policies or practices that restrict access based on many of the factors listed, and limited openings in transitional housing programs, these barriers make accessing safe and stable housing a formidable challenge. Shelter is itself a critical element of safety (Nyamathi et al. 2000), and permanent, safe housing solutions are needed.

The association of higher levels of education (college or graduate degree versus high school degree or GED) with a greater risk of experiencing weapon-involved violence runs contrary to prior research (Wong et al. 2016). Only 33 women in the sample reported education levels beyond high school, and we caution against further interpretation without more evidence.

## Limitations

The results from this study should be interpreted with consideration of study limitations. This study identified risk factors for gun and other violence, and causal relationships should not be drawn from the results, especially considering that the data are cross-sectional and time ordering of events can only be inferred from the wording of the questions. Additionally, not all potential confounders of the exposure-outcome relationships were available. For example, measures of childhood poverty and other factors known to be associated with the experience of both child and adult violence were not measured. As a result, the associations observed are likely confounded by other related factors.

Study measures are also subject to limitations common to survey research. For example, women may have underreported experiences of violence if exposure to violence has become so normalized it is not recognized as such (Huey 2015). Alternatively, recall bias could occur if women exposed to violence as adults are more likely to think about previous sources of violence, for example in childhood. It is also possible that some women are more willing to speak about experiences of violence, in childhood and adulthood, than others. Finally, the respondents ranged in age from 24 to 75 years old at the time of the interview; older respondents had to reach further back in their memories to recall childhood experiences of violence, which could result in measurement error.

The sample size is relatively small, resulting in wide confidence intervals on many measures of association. As a result, we cannot determine whether null results were due to a true lack of association or to the study being underpowered to detect differences of the observed magnitude. Additionally, we made multiple comparisons, which increased the possibility of observing a significant association due to statistical chance. Finally, there was significant overlap in the experience of each form of violence, and as such, separating the effects of each form from the others relies on fewer observations than desired.

## Conclusions

The prevalence of experiencing multiple forms of violence, and particularly gun violence, is consistently high across this and other studies of unhoused and unstably housed persons. National firearm violence statistics that shine light on the concentrated risk of violence and gun violence in economically vulnerable populations is critical to understanding the full extent of this persistent problem and to informing prevention efforts. Additionally, identifying and addressing maltreatment and associated factors in a comprehensive manner in childhood may yield far-reaching downstream benefits to the children involved.

## Data Availability

The datasets generated and/or analyzed during the current study are not publicly available due to the need to maintain confidentiality but are available from the PULSE team at the University of California San Francisco on reasonable request.
